# Developing a district level supportive supervision framework for community health workers through co-production in South Africa

**DOI:** 10.1186/s12913-021-06350-2

**Published:** 2021-04-14

**Authors:** Tumelo Assegaai, Helen Schneider, Vera Scott

**Affiliations:** 1 School of Public Health, University of the Western Cape, Cape Town, South Africa; 2 South African Medical Research Council Health Services to Systems Unit, University of the Western Cape, Cape Town, South Africa

**Keywords:** Community health workers, WBOT, Support, Supervision, Co-production, Participatory research

## Abstract

**Background:**

One of the key challenges of community health worker (CHW) programmes across the globe is inadequate supervision. Evidence on effective approaches to CHW supervision is limited and intervention research has up to now focused primarily on outcomes and less on intervention development processes. This paper reports on participatory and iterative research on the supervision of CHWs, conducted in several phases and culminating in a co-produced district level supportive supervision framework for Ward Based Outreach Teams in a South African district.

**Methods:**

Drawing on a conceptual framework of domains of co-production, the paper reflects on the implications of the research process adopted for participants, generation of research knowledge and recommendations for practice, as well as lessons for research on the supervision of CHWs.

**Results:**

Through the research process, participants reflected and engaged meaningfully, honestly and productively across hierarchies, and were able to forge new, dialogic relationships. The iterative, back forth feedback, involving a core group of participants across phases, enabled additions and validations, and informed further data collection. The culmination of the process was consensus on the key issues facing the programme and the generation of a set of recommendations for a local, context-specific framework of supportive supervision. The process of engagement, relationships built and consensus forged proved to be more significant than the framework itself.

**Conclusion:**

The co-production approach can enable local impact of research findings by providing a bottom-up collaborative platform of active participation, iterative feedback, knowledge generation and mutual learning that can complement guidance and frameworks from above. Although time consuming and not without its limitations, this approach to research has much to offer in advancing understanding of CHW supervision.

## Introduction

South Africa, like many other low and middle income countries, has adopted community health worker (CHW) programmes in the face of high chronic communicable and non-communicable disease burdens and human resources for health shortages [1, 2]. The country is also in the process of institutionalising universal health coverage, with the inclusion of CHWs as a component of Primary Health Care (PHC) [3, 4].

Despite their promise, community health worker programmes across the globe have experienced challenges that include limited training and resources, low trust with other primary health care workers and inadequate supervision [5–8]. Supportive supervision is considered among the key priorities for CHW programmes, and is required to nurture the skills, knowledge, confidence, and motivation of community cadres [9–12]. Supportive supervision is a process that promotes quality by strengthening relationships within the system, focusing on the identification and resolution of problems, and helping to optimize the allocation of resources for CHWs [13]. A systematic review conducted to inform the recent World Health Organization (WHO) guidelines on CHW programmes reported “very low certainty” regarding the evidence on supportive supervision [14].

Research on interventions to strengthen the supervision of CHWs has traditionally favoured experimental methodologies and systematic reviews of trials [14–18]. Supervision interventions, such as the use of mobile health technology and quality improvement strategies, are typically designed by researchers, sometimes in consultation with policy makers or programme managers. However, beneficiaries of these interventions, particularly at the coal face, are seldom directly involved in their design [19]. These studies fall under the umbrella of implementation science or knowledge translation research, which assumes that research findings deliberately packaged to ‘transfer knowledge’ are indeed accessed, understood and utilised by practitioners [20, 21]. However, Greenhalgh et al., as with others who have critiqued this notion, posit that knowledge “rarely conforms to this linear sequence”, and that the impact of research findings is limited to those who produce it [20–25].

Furthermore, intervention research on CHW supervision has up till now focused primarily on the outcomes of the interventions and less on their development processes. The literature is silent on how to develop CHW supportive supervision interventions that are participatory, and involve mobilisation of local tacit knowledge that can complement guidance and frameworks from research and policies; or how local stakeholders can be brought into research processes to develop and improve supervision interventions for CHWs.

This paper reports on 4 years of research engagement by the first author (as part of her PhD) in a South African district, culminating in a co-produced district level supportive supervision framework for Ward Based Outreach Teams (WBOTs - South Africa’s CHW Programme). Based on a deliberate research design, this process involved working in a participatory and iterative manner over time with relevant stakeholders at strategic and functional levels that can best be described as a co-production approach.

Co-production is a process of working together and building relationships between different groups of people to generate knowledge that coherently incorporates different viewpoints, as well as a ‘collaborative model of research that includes stakeholders in the research process’. [26, 27] Referred to as co-creation in some literature, co-production allows researchers to draw on the expertise of the practitioners to achieve a joint understanding, local innovation and context relevance [20, 28]. The co-production approach ensures impact of research findings by providing a platform for collaborative research with research users, through active participation and mutual learning [21–25]. This process also engenders a sense of value and importance by enabling research to be conducted with research users and not for them, by giving voice and by empowering otherwise silent frontline workers [20, 23, 24, 28, 29]. Key elements of co-production, as identified by Hickey et al., are sharing of power, including all perspectives and skills, respecting and valuing the knowledge of all those working together on the research, reciprocity and building and maintaining relationships [30].

Langley et al. [26] propose a framework of co-production that includes a set of principles and domains of influence of co-production. The principles draw on Greenhalgh et al’s work and include “using a systems perspective that acknowledges non-linearity and encourages local adaptation; positioning research as a creative enterprise that has human experience at its core; and emphasis on the process, the quality of relationships and applying facilitation techniques that consider power-sharing and utilise conflict as a positive force” [20]. Domains of influence (Table 1) operate at participant, knowledge and implementation levels [25].

**Table 1:** Domains of influence of co-production based on knowledge mobilisation

1. Influence on participants – creating the conditions for co-production	
2. Influence on knowledge – identifying and sharing knowledge for participants to learn practical implications of use	
3. Influence on implementation – combination of the influence on participants and knowledge allows for practical uptake and use of knowledge	

Supervision is a deeply relational process, embedded in social and professional contexts in the health system, that involves supervisors and supervisees at different levels of hierarchies across a range of functions and interactions [31]. A co-production approach to research is thus in keeping with a relational understanding of supportive supervision, where researchers do not formulate interventions in a top down manner, based on research findings, but rather seek to collectively generate knowledge [28].

The paper begins by describing the setting of the research, then lays out the conceptual framework of co-production as domains of influence adopted for the analysis and how the research unfolded in phases. This is followed by an exploration of the co-production process through the lens of domains of influence, and a discussion of the lessons for research on supportive supervision in CHW programmes.

## Methodology

### Setting

The study was conducted in Ngaka Modiri Molema (NMM) district, North West Province. The South African ward-based PHC outreach team (WBOT) functions at a ward level, where a group of six to ten CHWs provide basic preventive and promotive services on non-communicable diseases, HIV/TB and mother and child health at household and community level, supported and supervised by a professional nurse, called a team leader, and reporting to a PHC facility [32].

The North West Province was an early adopter of the WBOT programme after its launch in 2011, and recognised for its many achievements [33, 34]. However, from around 2014 onwards the programme started to experience difficulties, in the context of a wider fiscal and governance crisis in the provincial health system. During the course of the research (2016–20), the programme faced an increasing number of challenges related to sustained implementation of the programme. These challenges included a severe and growing shortage of team leaders, a halting of team leader induction training, inadequate support and supervision of team leaders, strained relations between WBOT members and PHC facility workers, and managers at district and provincial levels providing limited oversight and support to the programme [3, 10, 35]. Compounding these challenges, there were no official guidelines for supervision and support of WBOTs, in the province and country wide.

### Conceptual framework

**Fig. 1:**
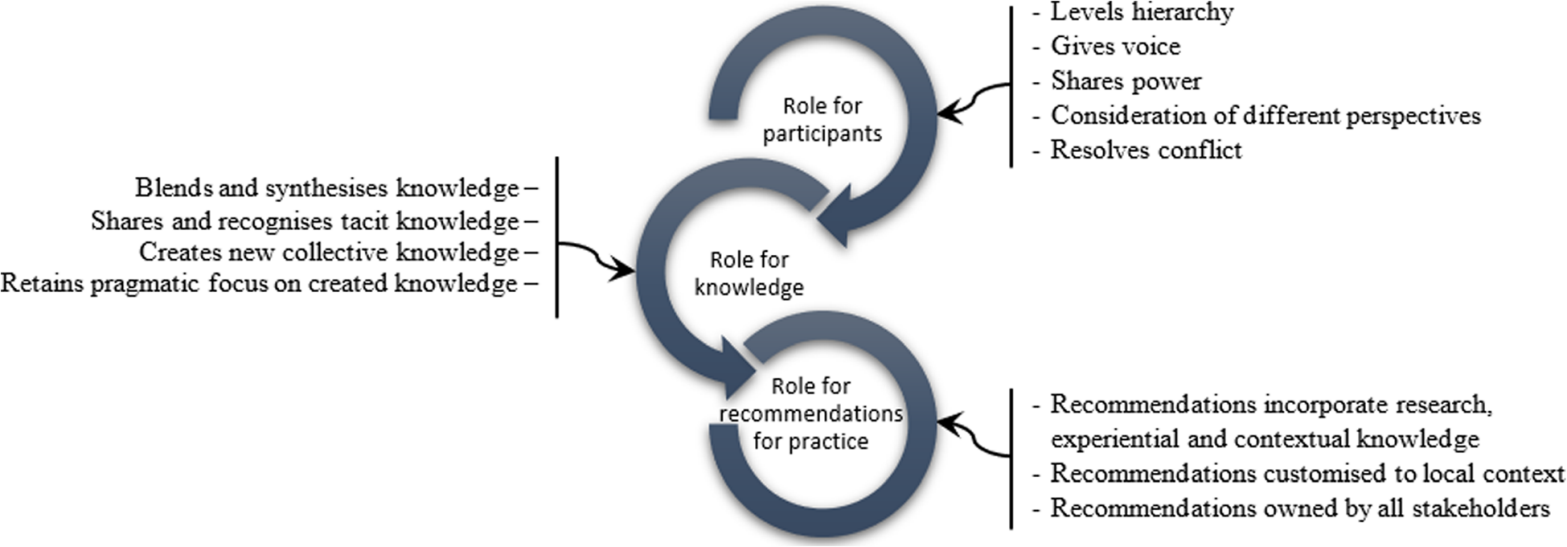
Roles of coproduction conceptual framework. Adapted from (Greenhalgh et al. [20]; Israilov & Hyung [28]; Langley et al. [25])

Drawing on theoretical understandings in the literature (summarised in Table 1), Fig. 1 outlines the approach to co-production in this paper, examining the role of co-production for participants, generation of research knowledge and recommendations for practice. The research design sought to bring together different categories of participants and give them voice to share knowledge and experiences, in an enabling and non-hierarchical environment. The approach recognised the value of shared understanding of experiences, while recognising that “*it is key in the early stages of building trust between diverse stakeholders and helps banish myths that constrain contextually sensitive solutions being developed*” [26]. The research aimed to generate collective knowledge and local, context-relevant recommendations owned by all stakeholders. This co-production process was made possible by the embedded nature of the researcher (TA), who is from the study area. She had previously worked in an non-governmental organisation supporting the health system, is knowledgeable about the local social context and able to communicate in seTswana (her mother tongue). TA developed the study design, collected the data and conducted the analysis of the research under the guidance of VS and HS.

### Co-production activities

**Fig. 2:**
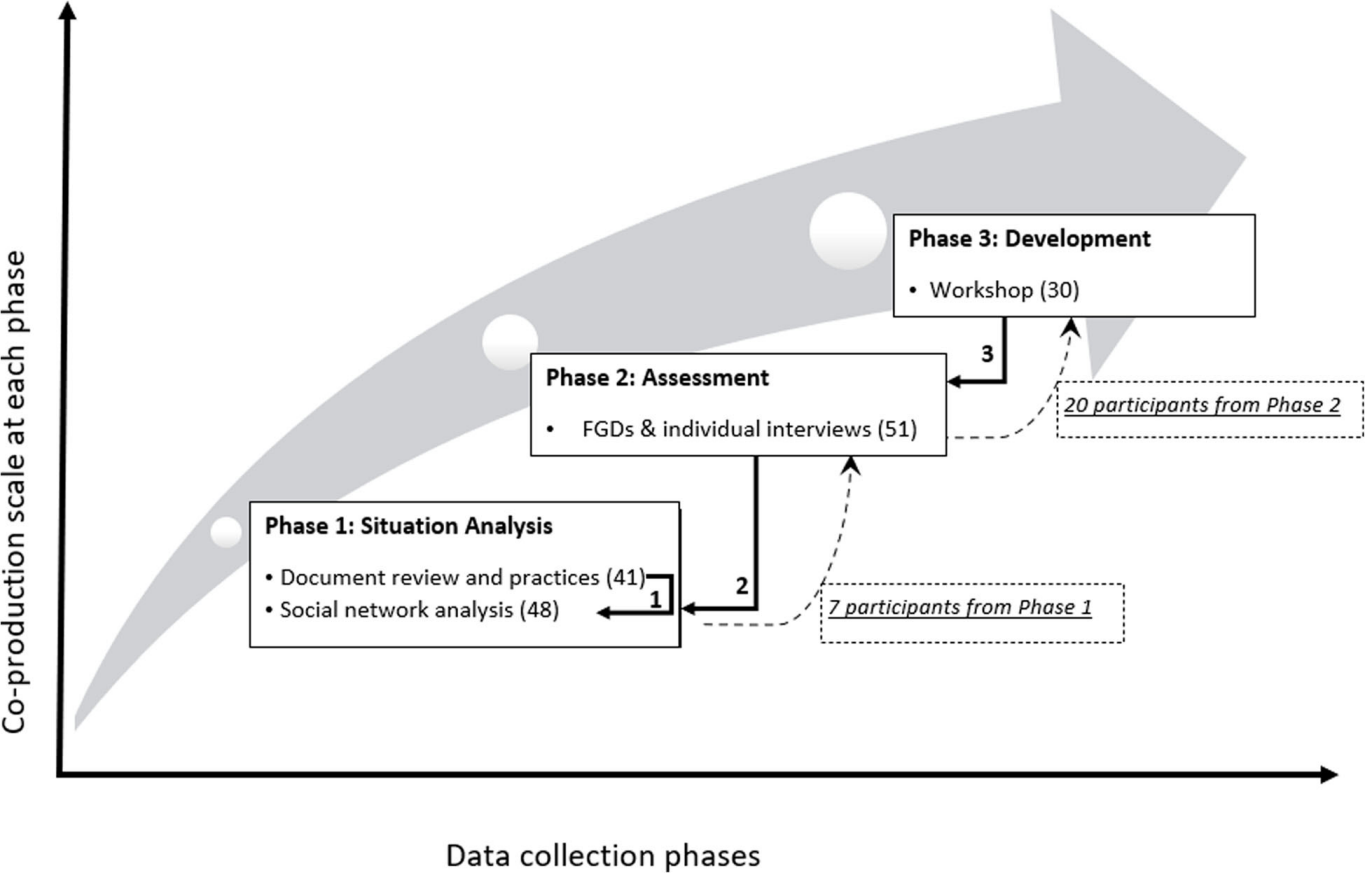
Co-production phases and data collection flow (numbers of participants in brackets)

This research involved a mix of quantitative and qualitative methods, undertaken in an iterative process in three phases, illustrated in Fig. 2 below. This included a situation analysis of policies, practices and relationships (Phase 1); an exploration of factors associated with trust in the supervisory relationships (Phase 2) and the development of a district level supervisory framework (Phase 3). The three phases aligned with the doctoral research objectives which sought to explore the mechanisms (inputs and processes) that could form the basis of supportive supervision of WBOTs, recognising that trust relationships were at the centre of a supportive supervision system.

In phase 1 (situation analysis), a qualitative, descriptive study that combined a document review of five available policy and guideline documents (nationally and provincially), and focus group discussions (FGDs) was conducted in NMM and a neighbouring district. A total of 41 PHC facility managers, team leaders and CHWs, purposefully sampled, participated in the FGDs. Themes and sub-themes that emerged from coded text in both the document review and the FGDs were thematically analysed, alignment across documents and practices analysed and strengths, weaknesses and gaps identified. The second part of phase 1 was a cross-sectional, quantitative study using social network analysis (SNA) in one sub-district of NMM district with the longest experience of WBOTs. Two of the three local areas in the sub-district with participants who had been associated with WBOTs since the early phases of the programme, were purposefully selected. The 48 participants in this phase were provided with feedback of the findings from the document review and preceding FGDs to validate and comment on the policies and practices of the WBOT supervision system (solid arrow 1, Fig. 2). Structured questionnaires surveyed the social and professional networks related to the WBOT programme using five questions that were representative of the three domains of supportive supervision. Sociographs of the WBOT supervisory system were generated. The data collection, analysis and findings of phase 1 are described elsewhere [31, 36]. Phase 2 was a qualitative, descriptive study involving specifically exploring trust relationships - workplace and interpersonal - in the district and primary health care supervisory system for WBOTs. Phase 2 was conducted in three of five sub-districts in NMM, among 51 participants (provincial managers, district managers, sub-district middle managers, focal persons, PHC facility managers, OTLs and CHWs) purposefully selected, with their respective facilities and WBOTs. In the sub-district where the prior social network analysis was conducted, seven participants were invited to participate in Phase 2 (bottom dashed arrow, Fig. 2). In this phase participants validated feedback of findings from phase 1 and further commented on the status and nature of relationships in the WBOT supervision system (solid arrow 2, Fig. 2). Audio recorded FDGs and individual interviews were conducted using a semi-structured interview guide and open-ended questions. The codes were identified and categorised deductively using thematic analysis. The data collection, analysis and findings of phase 2 are described elsewhere [37].

Based on the validated findings from Phase 1 and 2, phase 3 involved a workshop convened with a mix of practitioners [30] to agree on the elements of the WBOT supervisory framework. Participants, drawn from all sub-districts, the district office and provincial office, were selected by both the first author (TA) and the district. They included CHWs, team leaders, facility managers, district and provincial managers, with the researchers as the facilitators of the workshop. Twenty of the participants who had been part of previous phases of the study, identified for their insights, knowledge and different categories, formed part of the workshop (top dashed arrow, Fig. 2). The number of participants [30] was thought to be a reasonable balance between engagement and participation and meaningful generation of information. The venue for the workshop was jointly identified by the researchers and provincial managers.

The purpose of the workshop in phase 3 was to stimulate dialogue across disciplines, hierarchies and perspectives and was designed as a space which ensured maximum participation and dialogue of equals. Summaries of findings from the first two phases were prepared and sent to the participants (solid arrow 3, Fig. 2), with a workshop programme through email which was also distributed to the participants at the workshop.

An opening round at the workshop drew out the hopes and challenges of participants, and was followed by a brief presentation on the purpose and background of the workshop, supportive supervision concepts and summaries from the two earlier phases. Participants were then divided into four small working groups to discuss four broad themes identified from phase 1 and 2. All categories of practitioners were represented in each group, to draw out the different perspectives across levels. Participants deliberated on constraints and opportunities in each key area, presenting feedback and summative reflections from their group work to the plenary, followed by dialogue across groups through questions, answers, and comments. After these engagements, each group then developed three actionable strategies on their key area that would strengthen and form part of the WBOT supportive supervision framework in the district. The proceedings of the workshop were recorded, the ‘sticky’ notes contributed by participants were gathered, observations of the workshop dynamics were noted, and reflective notes by the two researchers (TA and VS) after the workshop were also recorded.

In sum, the co-production process framed questions in a manner that got participants to think in a certain way about the supervision of WBOTs and encouraged reflection at every stage, where researchers and participants learnt from each other in a dialogic process in iterative, back and forth engagements.

### Research ethics

Participants gave written consent to participate in all the studies, with attention paid to privacy and confidentiality. Information was also provided about the possibility of withdrawing from the study if they wished before the data were analysed. The study was approved by the University of the Western Cape’s Senate Research Committee and Ethics Committee, reference number BM17/3/3.

## Reflections on the co-production process

### Influence of co-production on participants

In this research, a sequential approach to participant groupings was adopted. In the first and second phases, FGDs were mostly conducted in separate categories: six with CHWs, four with team leaders and four with facility managers. Middle managers were interviewed individually in the second phase to avoid frontline health workers feeling intimidated by their supervisors.

However, one FGD in the second phase was deliberately set up as a mixed group of participants [7] across disciplines and facilities from the sub-district where the SNA was conducted. These individuals had participated in the preceding phase and were aware of the research topic and its importance. The idea here was to model engagements and dialogue across a hierarchy within a safe space. House rules were set at the beginning of the session to allow participants to listen and participate actively without any fear of intimidation. The researcher was constantly mindful of the power dynamics that could potentially play out, given the different levels in the hierarchy represented on the day. She frequently encouraged all participants to give their own unique reflections, and reassured them that the interactions and discussions were confidential. One participant reflected on the mixed group session by stating “*The idea of connecting personally, this is something we take for granted and never thought about. It is an opportunity to dig deep*”. Participants who appeared sceptical were specifically encouraged to express contrary views based on their experiences and observations. As one team leader commented, *“It has helped me grow emotionally and be sensitive to others”.*

In the final phase, an introductory session invited participants to reflect on their attitudes, hopes and challenges in the workplace, by responding anonymously to six questions on sticky notes. In this way participants were encouraged to provide open responses and voice opinions they would otherwise hold back if said verbally. For example, one participant in the workshop, sitting next to their supervisor, wrote “*I do not trust my workplace. I am not supported, not developed, intimidated, bullied*”. Such honest opinions were enabled by the iterative processes of presenting feedback on findings of previous phases to the same participants over time, and facilitating repeated reflections of their own local experiences. These sessions were a platform of learning and critical questioning of local practices on supervision and the WBOT programme. As one team leader reflected *“It just opened our eyes about things we were not taking seriously. It helped us a lot. We can see our gaps and what we are going to do to improve.”*

The researcher (TA) observed and kept notes of people’s reactions and participation through-out the research process. In the early stages of phase 1, CHWs were hostile and appeared angry with the process, the team leaders and the researcher. One team that had experienced difficulties with their payments, and had been without a team leader for some time, asked the researcher “*how does this (research) benefit us in the challenges we have?*”. Another group of CHWs, which had been a pilot team for the sub-district since the beginning of the programme expressed frustration at the lack of movement on career pathing or absorption into the health system. As the process unfolded, the CHWs from the two teams who participated in all the three phases were observed to express their challenges in a non-confrontational manner, and generally had good engagements with officials and managers from the district and the province. The ‘safe spaces’ of the research enabled useful and meaningful dialogue, with maximum participation by all players across hierarchies. By removing ‘social desirability’ constraints, more honest and productive reflections from a variety of perspectives were made possible. The participants were observed to mix comfortably in the breakaway groups, where everyone was given a chance to comment. CHWs led the feedback to the plenary workshop on behalf of some of the groups.

### Influence of co-production on knowledge generation

Inputs and comments that participants provided in the feedback sessions were treated as additions and validations of the findings. Moreover, they were used to inform data collection in the phases that followed, ensuring participants’ reflections were correctly captured while demonstrating that the knowledge generated was important and recognised. One manager, commenting on the SNA findings of limited communication between key individuals, acknowledged that “*You cannot nurture a relationship if you are not constantly in contact with one another. The PHC facility managers do not support team leaders*”. This serves as an example of how main points of discussion were carried through all the phases.

**Fig. 3:**
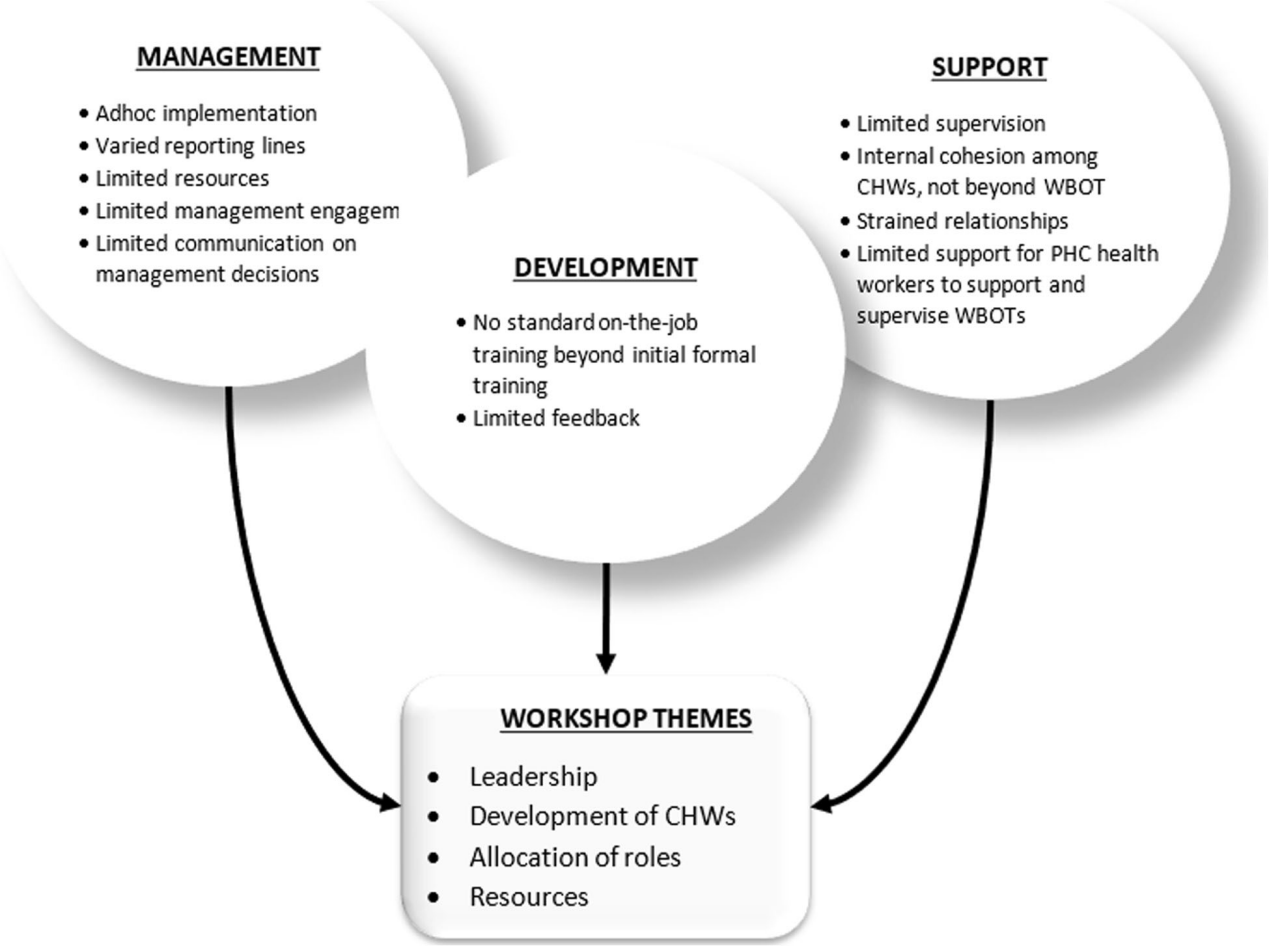
Findings of the study on the supervision of WBOTs

Phases 1 and 2 documented a number of weaknesses in both the design and practices of the supervisory system of the WBOTs. These are summarised in Fig. 3, categorised by the domains of supportive supervision (management, development and support). At the time of the research there was no official standalone framework guiding the supervision system of CHWs and WBOTs nationally or in the province. The absence of a clear guide on WBOT supervision led to varied reporting lines and practices of supervision. The critical challenges facing the programme also impacted on the supervision of WBOTs. A dire shortage of professional nurses in the province led to a shortage of team leaders. The other challenges included limited resources to undertake administrative and clinical tasks and inadequate engagement from middle and top management with the programme.

With regards to the development of WBOTs, supervisors (team leaders and PHC facility managers) lacked adequate orientation on the programme and supervision. Feedback on decisions relating to the programme, referrals from PHC facilities and general feedback on CHW performance was poor in many settings. In-service training for CHWs beyond the formal training was informal and infrequent. While there was a general sense of cohesion and support among CHWs themselves and with their team leaders, the interactions and relationships between WBOTs and PHC facilities workers was often strained. Because of poor engagement from management beyond PHC facilities, facilities had little support and encouragement in supervising WBOTs.

Drawing on these findings, four key themes were identified for the discussion in the final workshop: leadership, development of CHWs, allocation of roles and resources. Using the knowledge that was generated iteratively, and involving some participants in all the three phases, meant that by the time of the workshop there was general consensus on the key issues facing the WBOTs. This fed naturally into the development of a local framework that all could own.

### Influence of co-production on recommendations for practice

The culmination of the co-production process was the generation of a set of recommendations for a framework of supportive supervision as set out in Table 2. Participants in the final workshop brainstormed around possible strategies in response to constraints identified, and practicalities steps to solve challenges were discussed. An example was the issue of transport. There is a general shortage of state vehicles and almost none to the disposal of the WBOTs programme. Team leaders who are willing to make use of their vehicles are allowed to make an application to use their vehicles, however there is a limit on the kilometres they can claim for in a month and they are often requested to fulfil other tasks, like transporting medication for the facilities they are attached to. Options and suggestions in dealing with such limitations were explored by provincial managers, district officials and team leaders.

**Table 2:** WBOT supportive supervision framework

Theme	Constraints	Strategies
Development of CHWs	Formal training - Limited trainers for CHWs - Non-prioritisation of CHW training (Province) In-service training - Shortage of team leaders - Poor supervision from PHC facility workers - Lack of support from programme (HIV, MNCH, etc) managers	- In-service training must be done regularly by team leader (TL), facility manager, and peers - Human resource development should come with the schedule for training of CHWs on new guidelines and policies - Absorb existing CHWs into the health system
Allocation of roles	- Vacant posts - Severe shortage in key positions - Lack of supervision guidelines for the programme	- Appoint a fully functional WBOT (including TLs, CHWs, environmental health practitioners, data capturers, health promoters) - Develop supervisory tools for managers - Appoint PHC facility manager - Re-orientation of managers on the programme – in general role clarification - Training of CHWs - Training of newly recruited CHWs - Debriefing/early identification of burnout and act on it
Leadership	- Non-responsiveness of management to requests - Lack of resources - Lack of understanding of roles by managers - Lack of commitment by managers to the programme - Lack of capacity building	- Consistent implementation of the policies - Continuous support and interaction - Provision of resources e.g. working tools for TLs, CHWs uniform, name tags - Commitment, selflessness, passion - Good communication, confidentiality, equality - Training and development
Resources	- Shortage of team leaders and other relevant health workers - No transport for WBOT - Poor integration of WBOT into the health system, fragmentation - Limited space for administration work (office, stationery, medical supplies) - Lack of supplies (stationery, medical supplies)	- Create, fund and fill posts - Procure facility-based transport - Dedicated management structure for WBOT to be standardised - There must be a schedule for quarterly in-service training for CHWs TLs - Develop a framework for supervision

While some of the strategies of the framework remained at a fairly general level, the fact that these were collectively generated and owned, led to a discussion of implementation. The most significant output of the co-production process was thus not the framework itself, but rather the dialogue that enabled consensus on the problem and solutions. The process produced recommendations that were contextually relevant and tailored for the district, drawing from different perspectives, and which identified systemic issues and local opportunities. In addition, the process was able to model a supervisory approach that is supportive and inclusive.

## Discussion

Interventions, guidelines and reports on supervision of CHWs are typically designed or developed using an evidence-based approach, as reflected in the 2018 WHO guideline on health policy and system support to optimize community health worker programmes [14]. This paper reports on a doctoral process that piloted an alternative, participatory approach to developing a framework to improve supportive supervision for CHWs, which drew in all the role players and delivered a district-specific, actionable plan. From the onset, the research sought to be action oriented, by encouraging reflection through interviews, role modelling respectful relationships, and testing findings through iterative engagements over time using a co-production approach.

This research experience offers a number of lessons on co-production as an approach to supervision research.

Firstly, the time and effort that participants invest in generating knowledge makes ownership and uptake of recommendations for practice more likely [25]. However, as pointed out in the literature, the process is labour-intensive and time-consuming and conducting extensive co-production research may not always be feasible [20–23]. This research was conducted over 5 years, as data collection had to happen when the majority of participants were available, and findings from the preceding round of data collection needed to be ready and available for participants to comment on and validate.

Secondly, supportive supervision is a complex phenomenon that involves multiple actors and relationships and which therefore requires a systems perspective. The study involved participants at both functional and strategic levels in the WBOT supervision system. It demonstrated that co-production can be beneficial for social interactions as relationships across different hierarchies and functional levels are equalised and placed on a new footing [20, 23, 25, 38]. The research approach assisted in building confidence among participants and allowed them to articulate concerns and reflect honestly on their own local experiences, enabling a process of reciprocal accountability and consensus on the need to improve supervision for CHWs [39].

Thirdly, the research showed the importance of integrating and translating generic knowledge and recommendations on supervision within specific sets of relationships and context by mobilising tacit understandings and knowledge. The recommendations arising from the research cannot be regarded as an evidence based universal framework but rather the best fit for the local context. In this regard, the processes of engagement are more important as generalisable knowledge than the specific elements of the research product (i.e., framework).

Finally, the role and positionality of the researcher in the co-production process is key, and requires a high degree of reflexivity, and in some instances, resilience. Researchers may have to navigate reluctant participants, and entrenched ways of seeing, doing and engaging. It is also important to recognise that power relationships, priorities and expectations of researchers and policy makers inevitably “shape and direct these processes” [24, 26]. Linked to this, is a fact that researchers are not decision-makers and therefore may have limited influence, especially in the face of unfavourable contexts.

The study had some limitations that should be acknowledged. In particular, the wider provincial crisis, including civil servant protests and strikes in the Province during the study period delayed data collection process and affected the momentum of the co-production process. Despite these delays, a degree of stability was maintained, participation was secured over time and the planned data collection was eventually all completed. As indicated, the first author has had a prolonged engagement in the study site, and built on prior relationships during the research. This may have posed a potential bias in understanding and analysing findings. On the other hand, the author’s long association with the WBOTs programme enabled her to negotiate entry and participation and contextualise findings in trends over time. Another limitation was that the study was confined to supportive supervision in the formal PHC and the district health system and excluded communities where the services are rendered. The inclusion of communities in the study population and their contribution in the participatory process would have broadened knowledge generation and added valuable recommendations to the development of the framework.

## Conclusion

This research adopted a co-production approach to developing a district level framework for supportive supervision of CHWs, recognising that the phenomenon is fundamentally a system of relationships. Rather than seeking to develop technical recommendations using ‘evidence-based’ methodologies, it drew on the tacit knowledge of practitioners, modelling different behaviours, encouraging dialogic approaches, and working within and across groups to flatten hierarchies. Co-production can enable local impact of research findings by providing a bottom-up, collaborative platform of active participation, iterative feedback and mutual learning that can complement guidance and frameworks from above. However, this form of research is time consuming and not always feasible or without its limitations.

## Data Availability

The datasets generated and analysed during the study are not publicly available due to the qualitative nature of the research as this can potentially compromise participants’ identities, but codes are available from the corresponding author on reasonable request.

## Abbreviations

CHW: Community health worker
PHC: Primary Health Care
WHO: World Health Organization
WBOT: Ward-based outreach teams
NMM: Ngaka Modiri Molema
HIV/TB: Human Immunodeficiency Virus and Tuberculosis
FGDs: Focus Group Discussions
SNA: Social network analysis
MNCH: Maternal, Newborn And Child Health
TL: Team leader
